# Two Cases of Dropsy, in Which Unusually Large Doses of Digitalis Were Administered

**Published:** 1826-03

**Authors:** John Davy


ART. IV.—*Two Cases of Dropsy, in which unusually large Doses of Digitalis were administered.* By JOHN DAVY, M.D. F.R.S. &c.*

ASCITES.

CORNELIUS WOOD, first Veteran Company, aged thirty-seven; admitted 27th January, 1823. An Englishman; no trade; eighteen years in the service. Has never used mercury. Has served in the Mediterranean, in the Peninsula, in France, and in Canada. In 1814, while with his corps in Portugal, had an attack of fever, attended with mental alienation; for which he remained in hospital four months. He has also, for the last three months, been occasionally affected with tertian intermittent, and latterly with symptoms of pneumonia; for which he has been bled and blistered. Has used some sulphate of zinc pills for the ague.

* In a former Number, we briefly alluded to the occurrence of these cases: we now present two of them to our readers, extracted from the records at Chatham.—EDITORS.

He is now admitted with an anasarcous affection of the head, face, and lower extremities, and hydropic tumefaction of the abdomen. Bowels costive; cough, and expectoration; tongue white; skin pits.—℞. Pulv. jalap. comp. ʒj.

28th.—The powder operated briskly. There appears to be some tendency in the swelling to subside. *Pulse full.*—Venesectio ad ℥xij.

℞. Extract. conii, gr. xij.
Pulv. digitalis, gr. x.
Sodæ subcarbon. ʒss.
Infus. gentian, ℥iv. M. fiat mistur. quotidie sumend.

January 31.—A portion of his urine voided yesterday was examined. It contained a large proportion of mucus, and deposited much red sediment. It coagulated slightly, from the effect of heat and of nitric acid.

℞. Spirit. ætheris nitric, ss.

Vin. opii, gtt. xx.

Decoct. cinchonæ, ℥j.

Tinct. rhæi, ʒss. M. fiat haust. statim sumend.

℞. Extract. conii, gr. xij.
Pulv. digit. gr. xv.
Pil. hydrarg. gr. v.
Syrup. zingib. q. s. M. divid. in pil. vj. Capiat ij. omni nocte.

February 2.—Continues much the same. Has had no stool since last report.

℞. Sulph. magnes. ℥j.

Antimon. tartarisat. gr. j.

Vin. alœs, ℥j.

Infus. sennæ, ℥ij, M. Capiat ℥j. quaq. hora donec, &c.—Cont. mistura et pilulæ; omitt. haustus.

3d.—Vomited part of his mixture yesterday, but had afterwards several loose stools.—Cont. mistura sed augeatur. Pulv. digitalis ad gr. xv. in die.

4th. Had a severe attack of fever and ague yesterday. Has voided about three pounds of urine the last twenty-four hours.

℞. Decoct. cinchonæ,
Infus. gentian, ℥ij.
Tinct. sennæ, ℥ij. M. Capiat quotidie; et cont. mistura digit.

℞. Supertart. potassæ, ʒss. Divid. in chart. iv. sumat j. 3tia. quaque hora.

5th.—Complains to-day of difficulty of breathing and cough. Less urine; bowels loose. Rested ill.—Cont. medicam.

6th.—Says he is at present threatened with an attack of ague. Bowels loose; urine more copious. *Pulse sixty.*—Cont. med.

℞. Spirit. æther. nitr. ʒj.

Æther. sulphur. ℈j.

Vin. alœs, ℥j.

Aquæ menth. piper. ℥j. M. fiat haust. statim sumend.

9th.—Ague and fever very slight.—Cont. medicam.

11th.—Complains of nausea, and has a good deal of ptyalism.—Cont. sed aug. pulv. digit. ad gr. xx. Omit. pil. c. hydrarg.

14th.—Feels rather better. His mouth is still sore.

℞. Extract. conii, gr. xij.
Pulv. digit. gr. xxv.
Extract. taraxaci, ʒj. Fiat mass. et divid. in bolos No. viij. Capiat j. quaque hora.

17th.—Bowels rather confined. Says he has caught fresh cold. Some dyspnœa. Salivation diminished. Urine of a bright red colour, from purpurate of ammonia. *Pulse rather full.* He feels better in every respect.—Cont. med. Capiat pulv. rhæi ℈j. in infus. sennæ ℥ij.

21st.—Progressively improves; appetite pretty good; bowels regular. *Pulse soft.*—Cont. mistura et pilulæ ut antea.

22d.—Urine still high coloured; respiration more easy; bowels confined. *Pulse sixty-four, and rather full.*

℞. Pulv. rhæi, gr. xij.

Sodæ sulph. ʒij.

Infus. sennæ, ℥j. M.

23d.—Had three alvine evacuations since yesterday; urine continues high coloured. *Pulse soft;* tongue and skin moist; appetite good. Threw up a little coagulated blood yesterday, while coughing.—Venesection to ten ounces. Cont. med.; aug. pulv. digit. ad gr. xxx.

24th.—Feels better. *Pulse seventy-two, and soft;* bowels regular; blood taken buffed; urine not so high coloured; tongue moist; appetite good. Sleeps pretty well.—Cont. med.; et add gr. v. pulv. digit. ad misturam.

26th.—Bowels rather confined; in other respects better.

℞. Sodæ sulph. ʒij.

Infus. gentian, ℥jss.

Aquæ fontanæ, ℥jss. M.

29th.—Urine of nearly a natural colour; bowels quite regular; *pulse soft and natural;* tongue and skin moist; appetite good. When he lies on his left side, he is seized with a troublesome cough, but has no pain.—Cont. med.; aug. digit. ad gr. x.*

---

* This addition appears to have made the quantity of digitalis administered in twenty-four hours equal to *seventy grains;* viz. 45 in the mixture, and 25 in the form of pill or bolus.—See Dr. Davy's paper in our Number for June 1824, in which he states that he has given to the extent of 105 grains in twenty-four hours: the same digitalis, when given to other patients, producing its usual effects.—Editors.

March 2d.—Progressively improves. Bowels regular; appetite good; sleeps well.—Cont. med. ut antea.

4th.—Urine of nearly a natural colour; bowels regular; appetite good. Swelling quite gone.—Cont. med.

7th.—Daily improves. Bowels regular; appetite good.—Cont. med.

10th.—Progressively gains strength; look improved. Bowels regular; appetite good.

11th.—Convalescent.—Omit. med.

12th.—Discharged.

---

### ANASARCA.

June 11th, 1823.—Samuel Totton, 9th Lancers, aged thirty-four, admitted last night, having arrived from Weymouth two days ago; an invalid. He is a native of Lancashire, a hatter by trade; has been seventeen years in the 9th Light Dragoons. He served at Walcheren, in Portugal, Spain, and in France. Had fever at Walcheren, and has been subject to relapses occasionally ever since. He never had venereal disease. Patient strong made, and apparently of full habit. Says he fractured his knee eight months ago, while employed in the revenue service on the coast of Dorsetshire, near Bridport, by falling into the rift of a cliff; and, in consequence of this injury, has been ever since in hospital. On examination of his right knee, it appeared as if the inner condyle of the femur had been fractured, or otherwise injured. He has not the power of properly either bending or extending the leg, and complains of constant pain in the joint. About three months ago, the anasarcous symptoms made their appearance. He caught cold by pumping on his knee: an attack of ague was the consequence; and afterwards the dropsical affection followed. When he left Weymouth, on the 28th ult., he was but little swollen; but it has considerably increased since he commenced the march. Legs and thighs now much swoln and œdematous; face swoln. On examining the abdomen, fluctuation is distinctly felt. Pulse full, ninety-two; breathing short and difficult; pain across the chest; bowels irregular, now open; skin moist; sleeps badly; slight hard cough.—Habeat solut. potass. supertart. pro potu comm. Venesectio ad ℥xij.

June 12th.—Patient's breathing relieved by the bleeding; coughs less; feels generally better; blood scarcely buffed; much serum; urine scanty.

13th.—Pulse ninety, soft and full; bowels open; skin moist, temperature natural; swelling of the extremities subsided; slept badly. Feels better; urine more copious.

℞. Pulv. digitalis, gr. xv.

Pottassæ supertart. ʒss.

——— tartrat. ʒiv.

Syrup simp. ℥ij.

Aquæ hordei, ℥xiv. M. Capiat in dies.

June 14th.—Frequent stools during the night. Skin soft and moist; *pulse soft, ninety-six.* Complains of tightness across the chest.—Repet. mistura digitalis, &c. Omit. solut. potassæ supertart.

15th.—Says he feels better. Three stools only yesterday, *Pulse ninety, with some resistance;* skin moist, temperature equable; appetite improving.

16th.—Says he is better; looks improved. Two stools yesterday. *Pulse eighty, slightly resisting;* skin soft and moist; temperature equable; appetite good. Tightness across the chest continues. The mixture nauseates. Urine copious and free.—Mittatur sang. per scarificat. thorac. ad ℥iv.

℞. Pulv. digitalis, gr. xv.

Potassæ tartrat. ʒij.

——— supertart. ʒss.

Sodæ carbon. ʒij.

Syrup simp. ℥ij.

Decoct. hordei, ℥xiv. Capiat in dies.

17th.—Was relieved by the cupping on the chest. Breathing now more free. Two stools yesterday. No nausea; urine copious; *pulse seventy-five, soft and full;* skin soft and moist, temperature slightly raised; tongue furred; appetite tolerable.

18th.—Patient's state much as yesterday. *Pulse quicker, more full, and resisting.* Sleeps badly.

19th.—Patient had an exacerbation of fever last evening, when his *pulse was ninety-six, and resisting;* temperature raised, skin inclining to dryness; tongue loaded; anxiety and uneasiness; sleep disturbed. Now apyrexial; *pulse seventy-six, weak, and contracted.* Frequent ineffectual stools during the night; skin and temperature natural; anxious and uneasy; tongue furred, moist; urine free.

℞. Ol. ricini, ʒv.

Extract. opii, gr. ss.

Aquæ menth. piper. ℥j. M. Stat. sumend.

20th.—No considerable exacerbation last evening. The oil opened the bowels; rested badly; frequent startings. Complains now of great pain in the right lumbar region, extending towards the regio umbilicalis. Pain increased by pressure. *Pulse seventy-two;* tongue loaded; slight thirst; no appetite.—Mittatur sang. per scarificat. part. affect.

21st. The cupping relieved the pain. Passed a tolerable night, and feels better. Tongue furred; *pulse seventy-six, with*

*a jerk;* thirst; temperature rather above; skin moist. No stool since the operation of the oil; urine free. There is a fluctuation within the abdomen; but the extremities are reduced to their natural size.

℞. Pulv. rhæi,

Magnesiæ carbon. āā ℈j.

Aquæ menth. pip. ℥jss. M. fiat haustus stat. sumend.

22d.—Says he is rather better. Bowels evacuated. *Pulse eighty, full and hard;* skin rather dry; temperature above the natural standard; tongue loaded; sleep disturbed; slight thirst; appetite bad; urine free and copious, dark brown colour, with much sediment: it is serous, and coagulates with the application of heat or nitrous acid.—Venesectio ad ℥xij.

23d.—Blood buffed, and slightly cupped; bowels open. Says he feels better. Sleep less disturbed; skin soft, temperature natural, *pulse eighty, less resisting;* urine copious. Appetite continues to improve.

℞. Liq. ammon. acet. ℥ij.

Decoct. hordei, ℥viij. M. Capiat in dies.

Omitt. mistura cum pulv. digitalis.

24th.—Says he is still better. Had a keen appetite this morning. *Pulse eighty-four, soft and regular;* skin moist; bowels open thrice yesterday; urine copious and free; tongue nearly clean.

℞. Potassæ supertart. ʒss.

Pulv. zingib. ʒss. M. divide in pulv. no. iij.

Capiat unam ter quaterve in dies.

25th.—Patient easy, and feels better. Tongue moist, and nearly clean; pulse eighty-four, soft and regular; skin soft; bowels regular; urine copious, high coloured.—Repet. mistur. et pulv. potassæ supertart.

26th.—Four loose stools yesterday, and two during the night. Pulse eighty, slightly resisting; tongue moist and clean; skin soft and moist; urine free; appetite improving.

27th.—Slight pyrexia. Pulse eighty-two, compressible; temperature natural, skin soft; tongue nearly clean; bowels loose; urine free, colour natural. Says he feels better.

29th.—Slight pyrexia. Pulse eighty-four; tongue foul; skin soft, temperature raised; bowels open; urine free. Complains of acute pain in the left ear.—Applic. hirudines no. iv. post aurem.

30th.—Slight pyrexia. Skin soft, temperature slightly raised; pulse eighty-four, resisting; bowels open; urine free; ear relieved. Some œdema of the face and hands.—Cont. in usu misturæ et pulv. potassæ supertart. ut antea.

℞. Pulv. cinchonæ ℥ss. divide in pulv. iv. Capiat in dies.

July 5th.—The œdema and swelling have disappeared from every part. Apyrexia. Pulse eighty-six, soft; temperature natural; tongue clean; bowels open; urine free; health and appetite improving.—Repet. pulv. supertart. potassæ ut antea. Repet. pulv. cinchonæ et mistura ut antea.

9th.—Patient gradually improves; strength daily increasing; appetite good. Makes no complaint.—Repet. omnia.

12th.—He is now better than he has been for many months, and continues to improve. Makes no complaint. No appearance of œdema or swelling of the abdomen. Pulse eighty, regular; temperature natural, skin soft; bowels open; appetite good. Gains strength daily.

℞. Pulv. cinchonæ, ℨij.

Potassæ supertart. ℨiij.

Pulv. zingiber. ℈ss. M. divide in chart iij.

Capiat in dies.

19th.—He has continued to improve in health since last report. He is now free from complaint. Discharged, to appear before a Chelsea Board.

